# Total laparoscopic multi-compartment reconstruction via single-port lateral suspension: a novel mesh configuration based on the integral theory

**DOI:** 10.1093/jscr/rjag477

**Published:** 2026-06-19

**Authors:** Xiaoyue Yang, Zunsheng Zhang, Han Qin, Xinyu Cao, Jinyan Yuan, Jun Wang

**Affiliations:** Department of Gynecologic, The International Peace Maternity and Child Health Hospital, School of Medicine, Shanghai Jiao Tong University, Shanghai 200030, China; Shanghai Key Laboratory of Embryo Original Diseases, Shanghai 200030, China; Department of Gynecologic, The International Peace Maternity and Child Health Hospital, School of Medicine, Shanghai Jiao Tong University, Shanghai 200030, China; Shanghai Key Laboratory of Embryo Original Diseases, Shanghai 200030, China; Department of Gynecologic, The International Peace Maternity and Child Health Hospital, School of Medicine, Shanghai Jiao Tong University, Shanghai 200030, China; Shanghai Key Laboratory of Embryo Original Diseases, Shanghai 200030, China; Department of Gynecologic, The International Peace Maternity and Child Health Hospital, School of Medicine, Shanghai Jiao Tong University, Shanghai 200030, China; Shanghai Key Laboratory of Embryo Original Diseases, Shanghai 200030, China; Department of Gynecologic, The International Peace Maternity and Child Health Hospital, School of Medicine, Shanghai Jiao Tong University, Shanghai 200030, China; Shanghai Key Laboratory of Embryo Original Diseases, Shanghai 200030, China; Department of Gynecologic, The International Peace Maternity and Child Health Hospital, School of Medicine, Shanghai Jiao Tong University, Shanghai 200030, China; Shanghai Key Laboratory of Embryo Original Diseases, Shanghai 200030, China

**Keywords:** laparoscopic lateral suspension (LLS), triple-compartment prolapse repair, Denonvilliers fascia fixation, transvaginal mesh (TVM), alternative single-port pelvic reconstruction

## Abstract

This video article demonstrates a novel single-port laparoscopic lateral suspension (LLS) technique for concurrently correcting anterior, middle, and posterior pelvic organ prolapse (POP). The method utilizes a tailored mesh with fixation to Denonvilliers fascia to enhance posterior support, addressing a key limitation of traditional LLS. Compared to transvaginal mesh, this approach aims to reduce complication rates while offering a shorter learning curve for surgeons proficient in laparoscopy. We present the case of a 72-year-old woman with stage IV POP who underwent the procedure, which involved precise dissection to key anatomical landmarks and the creation of a peritoneal tunnel for mesh suspension. The conclusion is that this technique achieves comprehensive multi-compartment reconstruction with enhanced posterior support and preserved vaginal integrity, presenting a viable alternative to existing surgical options.

## Introduction

Pelvic organ prolapse (POP) is a common condition that often requires surgical intervention for symptomatic relief. The ‘Integrated Theory Paradigm’ (ITP) emphasizes the importance of addressing the pelvis as a functional unit, often necessitating repair of the anterior, middle, and posterior compartments simultaneously. Traditional vaginal mesh implantation (TVM) has been associated with high complication risks, including pain, infection, and mesh exposure, leading to a steep learning curve [1, 2]. As a safer alternative, laparoscopic lateral wall suspension (LLS) offers advantages such as reduced bleeding, the option for uterine preservation, and fewer sacral complications. However, a significant limitation of standard LLS is its higher recurrence rate for posterior pelvic defects, reported at 4.8% [3–6]. This video article (Video 1) introduces an innovative single-port laparoscopic technique that integrates posterior wall reinforcement via Denonvilliers fascia fixation, designed for severe POP cases requiring comprehensive, three-level pelvic floor reconstruction.

## Case report

A 72-year-old postmenopausal woman presented with a 10-year history of vaginal mass (enlarged from pigeon- to duck-egg size), lower back pain, urinary urgency (3–5 nightly voids), and constipation (3–5 bowel movements/day). POP-Q scores revealed Aa +3, Ba +6, C + 7, Ap +3, Bp +5, D + 6, indicating IV-degree uterine prolapse with anterior/posterior vaginal wall defects. Preoperative ultrasound confirmed a 36-mm uterine descent below the pubic symphysis and a 25 cm^2^ anal sphincter hiatus. Comorbidities included grade 1 hypertension and chronic nephritis.

The surgical procedure was performed as follows (as shown in Video 1):

**Exposure:** The procedure begins with adhesiolysis to release pelvic adhesions, followed by repositioning the small intestine to the right abdominal cavity. In single-port laparoscopy, the sigmoid colon is retracted superiorly and its mesentery secured to the left lower abdominal wall to optimize visualization.

**Bladder-vaginal space separation:** A uterine elevator elevates the uterus to flatten the anterior vaginal wall. Using an ultrasonic scalpel, the peritoneum at the bladder-uterine fold is incised, and the bladder-vaginal space is dissected bluntly and sharply. The bladder is retracted downward, creating a 3 × 7 cm space for mesh reinforcement. The posterior peritoneum of the broad ligament is punctured in avascular regions.

**Rectovaginal space separation:** The rectovaginal space is dissected from the posterior pelvis, avoiding Denonvilliers fascia. The plane is extended to the pelvic diaphragm fascia, identified by its white fibrous tissue, to accommodate posterior mesh placement.

**Figure 1:**
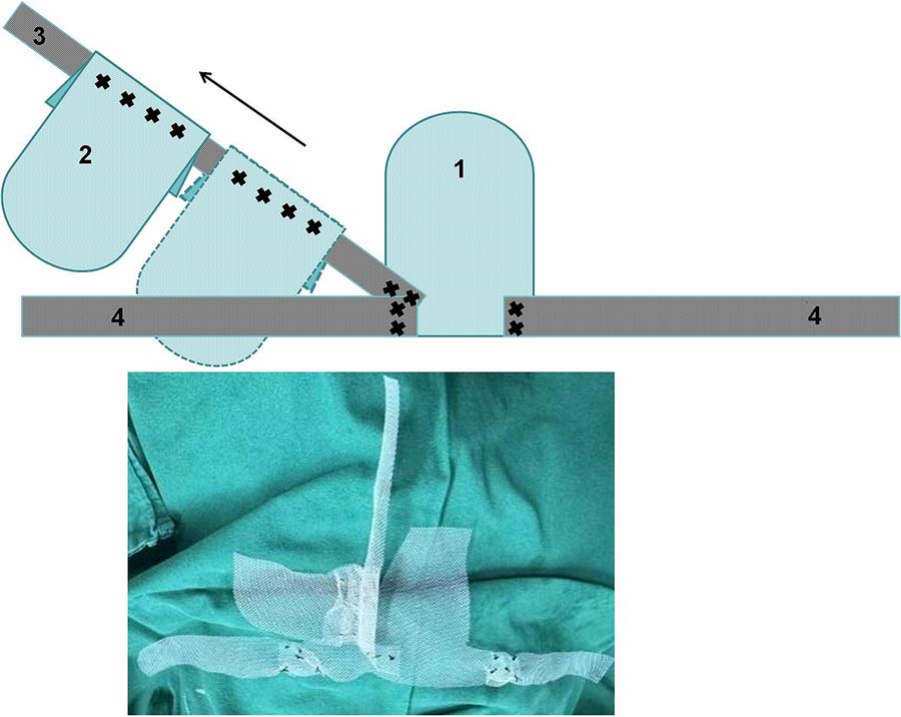
Artificial mesh pattern diagram and actual diagram.

**Mesh cutting and fixation:** A 15 × 15 cm TiLOOP®polypropylene mesh is tailored into four components: anterior/posterior vaginal wall segments, cervical arms, and lateral suspension arms. The anterior mesh (3 × 7 cm) (number 1 part in Fig. 1) and posterior rectovaginal branch (3–4 × 7–8 cm) (number 2 part in Fig. 1) are secured to the vaginal wall with absorbable sutures (two rows of four sutures) and to the bladder-cervical ligament/cervical isthmus with non-absorbable sutures (as shown in Fig. 1). The mesh is centered in the rectovaginal space, anchored at the Denonvilliers fascia apex, and fixed to the uterus via non-absorbable sutures.

**Peritoneal tunnel puncture:** A 4 cm incision is made above the anterior superior iliac spine. A subcutaneous tunnel is created at 40°–45° to the circular ligament, avoiding iliac vessels. Mesh ends (number 3 and 4 parts in Fig. 1) are pulled to 6 cm vaginal height. Subcutaneous sutures secure the mesh and peritoneum.

**Suturing:** Continuous absorbable sutures close the pelvic peritoneum to minimize adhesions.

## Discussion

The surgery was completed successfully without intraoperative complications or conversion. The patient had an uneventful postoperative recovery and was discharged in stable condition. During follow-up, her vaginal bulge, lower back pain, urinary urgency, nocturia, and constipation were substantially improved. At 6 months postoperatively, pelvic examination and POP-Q assessment confirmed satisfactory anatomical correction of the anterior, apical, and posterior compartments, with no evidence of recurrent prolapse. No mesh exposure, infection, mesh-related pain, urinary retention, or other late complications were observed. The patient reported high satisfaction and improved pelvic floor-related quality of life. This video demonstrates a single-port laparoscopic technique addressing anterior, middle, and posterior POP through three-level support (vaginal apex, mid-vagina, distal vagina). Unlike traditional laparoscopic lateral suspension (LLS) with a 4.8% recurrence rate for posterior defects [7], this method integrates a polypropylene mesh (3 × 7 cm anterior; 4 × 8 cm posterior) anchored to the Denonvilliers fascia apex and cervical isthmus, enhancing rectal and vaginal wall support [8]. Key innovations include: (i) Dynamic tension adjustment​ via sliding mesh branches (±1.5 cm). (ii) Three-dimensional reconstruction via precise dissection to the urethral-bladder junction and perineum. (iii) Reduced complications (0% mesh exposure vs. 4.8% in TVM) and shorter learning curves for laparoscopic-skilled surgeons. Postoperative outcomes show 94% anatomical success at 6 months, with POP-Q scores ≤ − 1 and improved PFDI-20 scores (68.5 → 22.3). This technique appears feasible and may enhance posterior support. Larger prospective studies are needed to evaluate safety, efficacy, and reproducibility.

## Supplementary Material

MyVideo_2_rjag477

## Data Availability

The data that support the findings of this study are available in the supplementary material of this article.
